# High carriage rate of high-level penicillin-resistant *Streptococcus pneumoniae *in a Taiwan kindergarten associated with a case of pneumococcal meningitis

**DOI:** 10.1186/1471-2334-5-96

**Published:** 2005-11-01

**Authors:** Tsai-Ling Lauderdale, Wei Yang Lee, Ming Fang Cheng, I Fei Huang, Yu Chen Lin, Kai Sheng Hseih, I-Wen Huang, Christine C Chiou

**Affiliations:** 1 Division of Clinical Research, National Health Research Institutes, Zhunan, Taiwan; 2 Kaohsiung Municipal United Hospital, Kaohsiung, Taiwan; 3 Department of Pediatrics, Veterans General Hospital-Kaohsiung, Kaohsiung, Taiwan; 4 National Yang-Ming University, Taipei, Taiwan

## Abstract

**Background:**

The Taiwan^19F^-14 *Streptococcus pneumoniae *clone and its variants are being found with increasing frequency in the Asia-Pacific region. A 5-year old child *with S. pneumoniae *meningitis caused by a high-level penicillin resistant strain (MIC = 4 μg/ml) was admitted to a hospital in southern Taiwan. We carried out a study to determine the potential source of this strain.

**Methods:**

Nasopharyngeal cultures were obtained from all children attending the same kindergarten as the index case. To determine their relatedness all isolates were compared by serotype, antimicrobial susceptibility profile and pulsed field gel electrophoresis (PFGE).

**Results:**

A high proportion of the children including the index case (32/78, 41.0%) carried *S. pneumoniae *in their nasopharynx (NP). The most common serotype was 19F (13/32, 40.6%). The PFGE types of the 19F serotype isolates obtained from the patient's blood, CSF and NP were identical and were related to 11 other serotype 19F NP isolates including 10 that were indistinguishable from the Taiwan^19F^-14 clone. All 14 isolates had similar high-level penicillin and multi-drug resistance. The serotypes of the other 19 NP isolates included 6A (2), 6B (10), 23F (5), 9V (1) and 3 (1). The overall rate of penicillin resistance in these *S. pneumoniae *from these children was 87.5% (28/32), with an MIC_50 _of 2 and MIC_90 _of 4 ug/ml. In addition, multi-drug resistant-isolates (isolates resistant to 3 different classes of antimicrobials) accounted for 87.5% (28/32) of all isolates.

**Conclusion:**

The high carriage rate of high-level penicillin- and multi-drug- resistant *S. pneumoniae *in a kindergarten associated with a case of pneumococcal meningitis emphasizes the need for restraint in antibiotic use and consideration of childhood immunization with conjugate pneumococcal vaccine to prevent the further spread of resistant *S. pneumoniae *in Taiwan.

## Background

There has been an alarming increase in recent years in the prevalence of penicillin- resistant *S. pneumoniae *and pneumococcal meningitis caused by penicillin non-susceptible *S. pneumoniae*. Thus far pneumococcal meningitis caused by high-level penicillin resistant strains (MIC = 4 ug/ml) accounts for only a small portion of the cases reported from various countries around the world [1-6]. A case of pneumococcal meningitis caused by a high-level penicillin resistant *S. pneumoniae *19F recently occurred in a 5-year old boy in southern Taiwan. The same microorganism was isolated from his CSF, blood and nasopharynx. We decided to determine whether the source of his infection might be kindergarten children with whom he had close contact. This was based on the knowledge that spread of multi-drug-resistant clones of *S. pneumoniae *occurs in the day-care center and kindergarten settings [7-10]. The most common clones include Spanish 23F, Taiwan 23F and 19F.

Nasopharyngeal surveillance cultures were performed on all the children who attended the same kindergarten as the patient. All the isolates of *S. pneumoniae *were characterized by susceptibility to a variety of antimicrobial drugs, serotype and pulsed field gel electrophoresis (PFGE) profiles.

### Case report

A 5-year-old boy with a two-day history of fever, vomiting and poor intake was brought to the emergency room of Veterans General Hospital-Kaohsiung, Taiwan in April 2002. He had cough for 2 days before the onset of fever and had visited a pediatric clinic, where oral antibiotics were prescribed. No otitis media or sinusitis was noted upon admission. He also had no prior hospitalizations or any major systemic illness in the past. On admission, his vital signs consisted of a temperature of 38.6°C (ear), pulse rate 108/min, respiratory rate 60/min and blood pressure of 134/87 mmHg. The physical examination revealed a drowsy child with nuchal rigidity and a positive Kernig sign. The Glascow Coma Scale was E1V1 M6. Chest radiography was normal. A lumbar puncture revealed an opening pressure of > 400 mmHg. The total white blood cell count of the CSF was 66/mm^3^, with 28% neutrophils, 68% lymphocytes, and 4% monocytes. The glucose and protein were 11 mg/dL and 596 mg/dL, respectively. Numerous gram-positive cocci in pairs were seen on microscopic examination. CSF and blood and nasopharyngeal cultures were positive for *S. pneumoniae*. The C-reactive protein was 12.3 mg/dL (normal <1 mg/dL). The immunoglobulin profile was within normal limits. The patient was intubated. Cefotaxime 200 mg/Kg/D and vancomycin 60 mg/Kg/D were administered immediately after lumbar puncture was performed. Dexamethasone 0.6 mg/Kg/D was also administered prior to the parenteral antibiotics and continued for 4 days. The isolates obtained from blood, nasopharynx and CSF demonstrated an MIC to penicillin of 4 μg/mL. A follow-up lumbar puncture was performed 72 hours after admission. The CSF glucose was 52 mg/dL and protein was 292 mg/dL with a lowered opening pressure. Vancomycin and cefotaxime were administered for a total of 15 days. He became afebrile on the 7th day. He was gradually weaned from the ventilator and fully recovered without neurological sequelae.

## Methods

### Nasopharyngeal cultures

Surveillance cultures of nasopharynx were performed on all the children who attended the same kindergarten as the patient. Nasopharyngeal culture was also performed on both of the parents and the younger sibling of the index case. Specimens were collected by a single investigator using a cotton swab placed 1–1.5 cm into the nasopharynx. The specimens were immediately inoculated on a 5% sheep blood plate (Becton Dickinson Microbiology System, Cockeysville, MD). All plates were incubated for 24–48 hours at 37°C in 5% carbon dioxide. *S. pneumoniae *was identified by typical colonial appearance, α-hemolysis, and gram stain. Confirmatory tests included optochin sensitivity and bile solubility tests (Becton Dickinson). All isolates were frozen at -70°C in tryptic soy broth for further analysis.

### Antimicrobial susceptibility testing

Minimum inhibitory concentrations were determined using the broth micro-dilution method following the guidelines of Clinical and Laboratory Standards Institute (formerly NCCLS) (CLSI/NCCLS) [11]. A final inoculum of 5 × 10^5 ^CFU/ml in Mueller-Hinton broth containing 2–5% lysed horse blood was used to inoculate the Sensititre STPF3 standard plate (Trek Diagnostics, East Essex, England). This device contained the following concentrations of antimicrobials (μg/ml): amoxicillin/clavulanic acid (2–16), cefepime (0.12–2), ceftriaxone (0.03–2), cefotaxime (0.12–4), cefuroxime (0.5–4), chloramphenicol (2 – 16), erythromycin (0.25–2), gatifloxacin (0.5–8), Gemifloxacin (0.03–0.5), levofloxacin (0.5–16), linezolide (0.25–4), meropenem (0.25–2), penicillin (0.03–8), moxifloxacin (0.25–8), tetracycline (0.5–8), trimethoprim/sulfamethoxazole (SXT) (0.5–4), and vancomycin (0.5–4). Interpretive criteria were based on those indicated in CLSI/NCCLS document M100-S14 [12]. In calculating resistance percentages, the 3 isolates from the patient were counted as one.

### Serotyping

Serogrouping and serotyping of *S. pneumoniae *were performed by the Quelling reaction using Omni serum, followed by pool, group, then factor serum (Statens Serum Institut, Copenhagen, Denmark).

### Pulsed field gel electrophoresis (PFGE)

Molecular typing of the genomic DNA was performed by PFGE. Preparation of DNA plugs and subsequent digestion by *SmaI *was performed following previously published protocols [13]. After staining with ethidium bromide, restriction fragments were imaged with an IS-1000 Digital Imaging System (Alpha Innotech Corporation, San Leandro, CA). PFGE patterns were analyzed using CHEF Mapper XA interactive software (version 1.2, Bio-Rad). International clones defined by the Pneumococcal Molecular Epidemiology Network (Spain^23F^-1, Taiwan^23F^-15, Taiwan^19F^-14, Spain^6B^-2) were used for comparison [14]. Cluster analysis was performed and dendrograms were prepared by the unweighted pair group method with arithmetic averages with the Jaccard coefficient. PFGE pulsotypes were assigned to clusters of isolates based on the published criteria [15].

## Results

**Figure 1:**
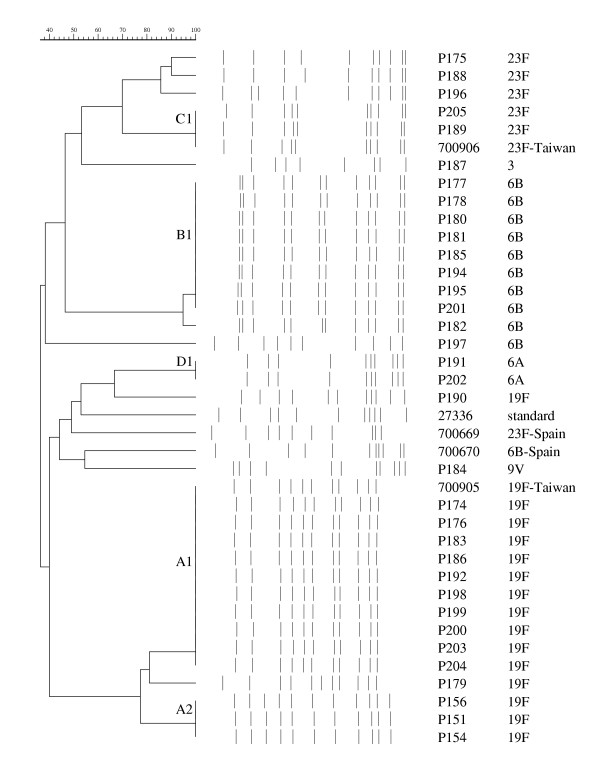
Dendrogram of 34 *S. pneumoniae *pediatric isolates (isolates starting with P) based on PFGE results. Reference strains: 27336, R6; ATCC 700669, Spain^23F^-1; ATCC 700670 Spain 6B-2; ATCC 700905 Taiwan^19F^-14; ATCC 700906 Taiwan^23F^-15.

**Figure 2:**
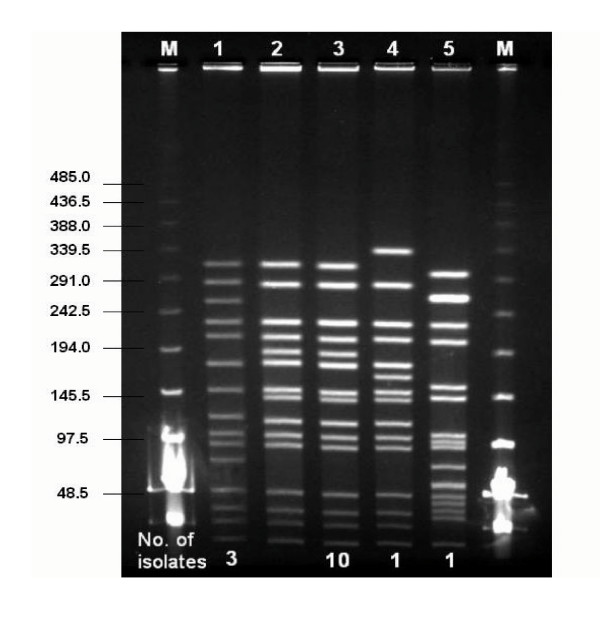
PFGE fingerprint patterns of *Sma*I restriction digest of serotype 19F *S. pneumoniae *isolates. M, lambda ladder molecular size markers (shown in kbp); lane 1, meningitis patient isolates; lane 2, ATCC700905 (Taiwan^19F^-14); lanes 3 – 5, other serotype 19F nasopharyngeal isolates from children attending the same kindergarten as the patient. Numbers at the bottom of the gel indicate the number of isolates with the same pattern.

*S. pneumoniae *was isolated from the nasopharynx of 32 (41.0%) of the 78 children who attended the same kindergarten, including the index case (Table 1). The age of the children in the current study ranged from 4 to 6.5 years old. None of the children in the kindergarten including the index case had received conjugate pneumococcal vaccine. No *S. pneumoniae *was found in the nasopharynx of the parents and the younger sibling of the index case. A total of 34 isolates were available for further analysis including the CSF, blood and nasopharyngeal isolates from the index case and 31 isolates from the nasopharynx of the other children attending the kindergarten. The most common serotype isolated from the nasopharynx was 19F (13/32, 40.6%). This was followed by serotypes 6B (10/32, 31.3%,) and 23F (5/32, 15.6%). There were two isolates of serotype 6A and one isolate each of serotypes 9V and 3. The isolates of *S. pneumoniae *recovered from CSF, blood and nasopharynx of the index case were identical by PFGE (Figure 1, A2 pulsotype). Ten of the 12 19F isolates from the other children were identical to the Taiwan^19F^-14 clone (Figure 1 A1 pulsotype, Figure 2 lanes 2 and 3). The isolates from the index case differed from the Taiwan^19F^-14 clone by 4 bands, indicating it was a variant of the Taiwan^19F^-14 clone (Figure 1 A2 and A1 pulsotypes, Figure 2 lanes 1 and 3).

**Table 1:** Distribution of serotypes, PFGE patterns and antimicrobial susceptibility profiles of 34 isolates of *S. pnuemoniae *isolates from 32 children attending a kindergarten in Kaohsiung, Taiwan

Isolate^a^	Serotype	PFGE type^b^	MIC (ug/ml) of:^c^											
			
			PEN	AUG	FRX	CRO	FTX	FEP	MEM	CHL	ERY	LEV	LID	SXT
P151^a^	19F	A2	4	4	>4	2	2	4	1	8	>2	1	1	>4
P154^a^	19F	A2	4	4	>4	2	2	4	1	8	>2	1	1	>4
P156^a^	19F	A2	4	4	>4	2	2	4	1	4	>2	1	1	>4
P174	19F	A1	2	≤2	4	1	1	0.5	0.5	8	>2	1	2	4
P176	19F	A1	2	≤2	4	1	1	1	0.5	4	>2	2	2	4
P183	19F	A1	2	≤2	>4	1	1	1	0.5	8	>2	1	2	4
P186	19F	A1	2	≤2	4	1	1	1	0.5	8	>2	1	2	4
P192	19F	A1	2	≤2	4	1	1	1	0.5	4	>2	2	2	4
P198	19F	A1	2	≤2	>4	1	1	1	0.5	8	>2	2	2	4
P199	19F	A1	2	≤2	>4	1	1	1	0.5	8	>2	1	2	4
P200	19F	A1	4	≤2	>4	1	1	1	0.5	4	>2	1	2	4
P203	19F	A1	2	≤2	>4	1	1	2	0.5	8	>2	2	2	4
P204	19F	A1	4	≤2	>4	1	1	1	0.5	4	>2	1	2	4
P179	19F		4	≤2	>4	1	1	1	0.5	4	>2	1	2	4
P190	19F		4	≤2	>4	2	2	2	0.5	4	>2	1	1	>4
P177	6B	B1	4	≤2	>4	1	1	1	0.5	4	>2	1	1	>4
P178	6B	B1	2	≤2	>4	1	1	1	0.5	4	>2	1	2	>4
P180	6B	B1	4	4	>4	1	1	1	0.5	4	>2	1	2	4
P181	6B	B1	4	4	>4	1	1	1	0.5	4	>2	1	1	>4
P185	6B	B1	4	≤2	>4	1	1	1	0.5	4	>2	1	2	>4
P194	6B	B1	2	≤2	>4	1	1	1	0.5	4	>2	1	2	>4
P195	6B	B1	2	≤2	>4	1	1	1	0.5	4	>2	1	2	>4
P201	6B	B1	4	≤2	>4	1	1	1	0.5	4	>2	1	2	>4
P182	6B		2	≤2	4	1	0.5	1	≤0.25	4	>2	1	2	>4
P197	6B		≤0.03	≤2	≤0.5	≤0.06	≤0.12	≤0.12	≤0.25	4	≤0.25	≤0.5	1	≤0.5
P189	23F	C1	2	≤2	>4	1	1	1	≤0.25	8	>2	1	2	≤0.5
P205	23F	C1	2	≤2	>4	1	1	1	0.5	8	>2	1	2	4
P175	23F		4	≤2	>4	2	1	2	0.5	8	>2	1	2	4
P188	23F		4	≤2	>4	2	1	2	0.5	8	>2	1	2	4
P196	23F		2	≤2	>4	2	1	1	0.5	8	>2	1	2	>4
P191	6A	D1	≤0.03	≤2	≤0.5	≤0.06	≤0.12	≤0.12	≤0.25	4	>2	1	1	4
P202	6A	D1	0.06	≤2	≤0.5	≤0.06	≤0.12	≤0.12	≤0.25	8	>2	1	1	4
P184	9V		4	4	>4	2	2	2	0.5	8	>2	2	1	>4
P187	3		≤0.03	≤2	≤0.5	≤0.06	≤0.12	≤0.12	≤0.25	4	≤0.25	2	1	≤0.5

^a ^Isolates P151, P154, and P156 were from blood, nasopharynx, and CSF of the meningitis patient, respectively. All other isolates were from nasopharynx of children attending the same kindergarten as the patient.

^b ^PFGE type, Pulse field gel electrophoresis pulsotype; see Figure 1.

^c ^AUG, amoxicillin/clavulanic acid; CHL, chloramphenicol; CRO, ceftriaxone; ERY, erythromycin; FEP, cefepime; FRX, cefuroxime; FTX, cefotaxime; LEV, levofloxacin; LID, Linezolid; MEM, meropenem; PEN, penicillin; SXT, trimethoprim/sulfamethoxazole. All isolates were resistant to tetracycline (MIC > 8 ug/ml) except isolate P187, which was susceptible (MIC ≤ 0.5 ug/ml). All isolates were susceptible to vancomycin (MIC ≤ 0.5 ug/ml).

All serotype 19F isolates (15 isolates from 13 children) were high-level penicillin (MIC ≥ 2 ug/ml) and multi-drug resistant. The three isolates from the case exhibited similar antibiogram as the other 12 serotype 19F nasopharyngeal isolates from his kindergarten contacts (Table 1). Although the 19F *S. pneumoniae *isolates exhibited similar antibiograms, the isolates from the patient were more resistant with higher amoxicillin/clavulanic acid, cefotaxime and cefepime MIC. In addition, based on meningitis interpretive criteria, isolates obtained from the index case were resistant to cefotaxime (MIC 2 ug/ml), cefepime (MIC 4 ug/ml), and ceftriaxone (2 ug/ml). The serotypes isolated from the other 19 children also exhibited high-level penicillin resistance except for serotypes 6A and a single strain of 6B, and 3. The overall rate of highly penicillin resistant *S. pneumoniae *(MIC 2 – 4 μg/ml) isolated from the nasopharynx of these 32 children was 87.5% (28/32). In addition, 13 (40.6%) of these 32 isolates had a penicillin MIC of ≥ 4 ug/ml. The rate of resistance to erythromycin, cefuroxime, trimethoprim/sulfamethoxazole and tetracycline were also high; at 93.8% (30/32), 87.5% (28/32), 90.6% (29/32) and 96.9% (31/32), respectively. Multi-drug resistant-isolates (isolates resistant to 3 different classes of antimicrobials) accounted for 87.5% (28/32) of all isolates. Most isolates remained susceptible to amoxicillin/clavulanic acid (81.25%, 26/32) and cefotaxime (84.4%, 27/32) despite the high prevalence of penicillin resistance. All isolates demonstrated universal susceptibility to all fluoroquinolones including gatifloxacin, gemifloxacin and moxifloxacin (data not shown) in addition to levofloxacin. All isolates were also uniformly susceptible to linezolid and vancomycin (Table 1).

## Discussion

Nasopharyngeal carriage of *S. pneumoniae *in day-care center attendees has been well documented [9,10,16,17]. Studies have found the carriage rates to range from 44%–65% at day-care centers, with one study reporting 53% of isolates highly resistant to penicillin, and another study reporting 13–19% of multi-drug resistance in serotype 14 isolates [16,17]. Taiwan has an extremely high carriage rate of penicillin-resistant *S. pneumonia*e among children attending day care centers and kindergartens [8]. The current report demonstrates an even higher nasopharyngeal carrier rate of penicillin resistant strains of 87.5% compared to 71.5% previously reported by our group [8]. In addition, 40.6% of the isolates in the present study had a penicillin MIC of ≥ 4 ug/ml, the highest percent of isolates with such high MICs reported in the literature [1-4,6]. The absence of resistance to fluoroquinolones is probably related to their uncommon use by pediatricians.

It is unclear why 41.0% of the children in the Kindergarten carried a variety of strains of *S. pneumoniae *yet only one child developed bacterial meningitis with serotype 19F. The child did not have history of recurrent infection and his immunoglobulin profiles were normal. It is unlikely that he had a congenital or acquired immunodeficiency or asplenia that predisposed him to invasive pneumococcal infection. Further studies are needed to determine if the invading strain is more virulent than the others. We have no ready explanation why the *S. pneumoniae *serotype 19F isolated from the index case exhibited a different albeit possibly related PFGE pattern from those isolated from his kindergarten contacts. The nasopharyngeal swab was collected at the same time as the CSF and blood cultures. We do not know how long the patient was colonized before the onset of his disease. However, it is well recognized that nasopharyngeal colonization precedes pneumococcal infection and studies have shown that *S. pneumoniae *strains recovered from the CSF of the meningitis patients to be the same as the strains carried in the nasopharynx of patients [18,19].

The Taiwan^19F ^clone is the most common serotype that causes pneumococcal diseases in Asian countries [20]. A recent study of *S. pneumoniae *isolates from patients with meningitis in Japan found high-level penicillin resistance to be associated with the presence of multiple (three) abnormal penicillin binding protein (*pbp*) genes. Serotype 19F was among those showing the greatest high-level penicillin resistance [6]. Another report from Japan found that the most prevalent serotype in a major Japanese medical center to be 19F, the majority of which were Taiwan^19F^-14 clone and its variants [21]. High-level antibiotic resistant strains of the Taiwan^19F ^clone and its variants are also relatively common in invasive and non-invasive pneumococcal infections in New Zealand [13,22].

The other serotypes isolated from children in the current study were similar to those encountered in our previous surveillance studies of day care centers and kindergartens in Kaohsiung, in which serotype 23F was the most common, followed by 19F, 6B, 6A, and 14 [23]. We were surprised by the absence of serotype 14, a serotype commonly found in nasopharyngeal and invasive isolates in children ≤ 5 years old, especially those ≤ 2 years old [3,4,24]. The differences between the current and previous studies in the distribution of serotypes may be accounted for by differences in the ages of the children. The children in the current study were older (4 – 6.5 years) compared to 2 months to 7 years in the prior study.

## Conclusion

*S. pneumoniae *and *Neisseria meningitidis *became the leading causes of meningitis in the United States and Netherlands after the widespread use of *H. influenzae *type b vaccine [5]. With the introduction of conjugate pneumococcal vaccine, there is now considerable evidence showing substantial decreases in invasive pneumococcal diseases and concomitant penicillin resistance in vaccine and vaccine-related serotypes in children who were vaccinated [25,26]. Although we did not find *S. pneumoniae *carriage in the parents and the young sibling of the index case, spread of *S. pneumoniae *and antibiotic-resistant serotypes from day-care center attendees to their siblings has been reported [17,27]. In addition, prior antibiotic use has been shown to contribute to increased *S. pneumoniae *colonization and diseases [16,17,28]. In February 2001 the Bureau of National Health Insurance in Taiwan implemented a new rule restricting antimicrobial prescription for acute upper respiratory infections in ambulatory patients. This has significantly decreased antimicrobial consumption in this country. Nevertheless, there are still an excessive number of ambulatory patient visits for respiratory infections resulting in antimicrobials being prescribed [29]. It is hoped that a combination of restraint in antibiotic use and implementation of childhood immunization with conjugate pneumococcal vaccine can reduce the burden of pneumococcal illness and multidrug resistant strains in Taiwan and other countries.

## Competing interests

The author(s) declare that they have no competing interests.

## Authors' contributions

TLL supervised the molecular study and antimicrobial susceptibility testing, carried out the analysis, and prepared the final manuscript. WYL assisted in specimen and data collection of the children attending the kindergarten, and identification and shipment of specimen. MFC, IFH, YCL, and KSH assisted in taking care of the patients and specimen collection. IWH carried out the molecular and antimicrobial susceptibility testing. CCC conceived and coordinated the study, carried out the specimen collection and analysis, and prepared the draft manuscript.

## Pre-publication history

The pre-publication history for this paper can be accessed here:


